# Outcomes of resection for colorectal cancer hepatic metastases stratified by evolving eras of treatment

**DOI:** 10.1186/1477-7819-9-174

**Published:** 2011-12-30

**Authors:** Kun-Ming Chan, Jy-Ming Chiang, Chen-Fang Lee, Ming-Chin Yu, Wei-Chen Lee, Jinn-Shiun Chen, Jeng-Yi Wang

**Affiliations:** 1 Department of General Surgery, Chang Gung Memorial Hospital at Linkou, Chang Gung University College of Medicine, Taoyuan, Taiwan; 2 Department of Colorectal Surgery, Chang Gung Memorial Hospital at Linkou, Chang Gung University College of Medicine, Taoyuan, Taiwan

**Keywords:** colorectal cancer, hepatic metastasis, liver resection, recurrent patterns, evolving eras

## Abstract

**Background and purpose:**

The outcomes and management of colorectal cancer (CRC) hepatic metastasis have undergone many evolutionary changes. In this study, we aimed to analyze the outcomes of patients with CRC hepatic metastasis in terms of the era of treatment.

**Methods:**

We conducted a retrospective review of 279 patients who underwent liver resection (LR) for CRC hepatic metastases. The prognoses of patients treated pre-2003 (era 1) and post-2003 (era 2) were examined.

**Results:**

Of the patients included in the study, 210 (75.3%) had CRC recurrence after LR. There was a significant difference in the ratio of CRC recurrence between the 2 eras (82.0% in era 1 *vs*. 69.5% in era 2; *p *= 0.008). Analysis of recurrence-free and overall survival rates also showed that the patient outcome was significantly better in the post-2003 era than in the pre-2003 era. Further analysis showed that a significantly higher percentage of patients in era 2 had received modern chemotherapeutic regimens including irinotecan and oxaliplatin, while patients in era 1 were mainly administered fluorouracil and leucovorin for adjuvant chemotherapy. Among patients with CRC recurrence, a significant ratio of those in era 2 underwent surgical resection for recurrent lesions, and these patients had a better survival curve than did patients without resection (34.1% *vs*. 2.2% for 5-year survival; *p *< 0.0001).

**Conclusion:**

The incidence of CRC recurrence after LR for hepatic metastasis remains very high. However, the management and outcomes of patients with CRC hepatic metastasis have greatly improved with time, suggesting that the current use of aggressive multimodality treatments including surgical resection combined with modern chemotherapeutic regimens effectively prolongs the life expectancy of these patients.

## Background

Hepatic metastasis is the most common form of distant spread of primary colorectal cancer (CRC) and occurs in over 50% of patients with metastases. Aggressive liver resection (LR), which provides the only curative treatment, is believed to have improved the long-term outcome of these patients [1-3]. Recently published studies have shown that these patients have variable 5-year survival rates ranging from 36% to 58% [4-8]. However, despite the excellent results of aggressive treatment for metastatic CRC, numerous patients still develop recurrence after LR for metastatic tumors.

Although the prognostic factors and scoring systems that determine patient outcomes after LR are well established,[4,9-11] most of the information regarding LR for metastatic CRC is from Western countries. However, CRC has become a common gastrointestinal malignancy and subsequently ranked as a leading cancerous disease in Taiwan and other East Asian countries during recent years. Thus, we gathered data and retrospectively reviewed our experience of LR for patients with hepatic metastasis from CRC. Additionally, since the treatment of metastatic CRC has changed greatly over the last decade, the patient cohort was grouped according to the timeframe of treatment to evaluate the evolution of outcome over the years.

## Materials and methods

### Patients

This study included 279 patients with CRC hepatic metastasis who underwent LR with curative intent between July 1988 and December 2008 at Chang Gung Memorial Hospital Linkou Medical Center (Taoyuan, Taiwan). The data in the medical records of these patients were retrospectively reviewed and analyzed for clinical characteristics, surgical management, and outcome upon approval of the institutional review board. Hepatic metastasis of CRC was confirmed for all patients by histological examination of specimens derived from LR. There were 177 men and 102 women, and their median age at the time of LR was 61 years (range, 21-88 years). The patient cohort was divided into 2 subgroups: the first 15-year period was defined as pre-2003; era 1, *n *= 128), and the later 5-year period was defined as post-2003; era 2, *n *= 151). The clinicopathologic features and outcomes of patients were compared between the 2 groups.

### LR for CRC hepatic metastases

Before surgery, all patients were thoroughly evaluated with appropriate imaging studies, including computed tomography (CT) of the abdominal and pelvic areas, chest roentgenography, and/or chest CT, to determine the clinical status of the CRC and hepatic metastasis. Positron emission tomography (PET) or PET/CT was not routinely performed, but were done for patients who had undergone equivocal conventional imaging studies to confirm advanced disease and, occasionally, to identify potentially resectable lesions. Resectability with curative intent required complete resection of all hepatic metastatic lesions and preservation of a sufficient volume of remnant liver. In particular, a patient with concurrent unresectable extrahepatic metastases was considered unsuitable for LR. LR was performed using either the surgical clamp-crush technique or the Cavitron Ultrasonic Surgical Aspirator (CUSA; Valleylab, Inc., Boulder, CO, USA). Inflow vascular control was not routinely applied during the transection of liver parenchyma. The extent of LR was defined according to Couinaud's classification of liver segments.

### Postoperative follow-up

After LR, patients were followed up and monitored for tumor recurrence by physical examination, measuring the serum carcinoembryonic antigen (CEA) levels, and conducting abdominal ultrasonography 1 month after surgery, and every 3 months thereafter. CT and/or magnetic resonance imaging of the chest, abdomen, and pelvis were performed at yearly intervals or whenever cancer recurrence was suspected. A PET or PET/CT scan was occasionally arranged for patients who underwent equivocal conventional imaging studies and experienced unexplained elevation of serum CEA levels after 2003. Perioperative chemotherapy mainly depended on tumor characteristics indicating aggressive disease, availability of chemotherapeutic regimens, patient's physical condition, and affordability of the chemotherapy drugs. Usually, postoperative adjuvant chemotherapy was recommended for all patients, unless a patient was unwilling to receive chemotherapy or a patient's physical status was unsuitable for chemotherapy administration. The chemotherapeutic options were mostly fluorouracil and leucovorin before 2003 and a combination of new options, including irinotecan, oxaliplatin, capecitabine, bevacizumab, and cetuximab, after 2003. Pyrimidine analogue capsules were prescribed for patients who had not received intravenous chemotherapeutic regimens. CRC recurrence was defined as the presence of a lesion that was histologically proven from either biopsy or surgical resection or the presence of a lesion that was detected by cross-sectional imaging studies and/or concurrent with an elevation in serum CEA level. Intrahepatic recurrence (IHR) was defined as tumor recurrence initially detected in the liver and no additional extrahepatic lesions detected within the following 3 months. Systemic recurrence (SR) was defined as initial recurrence found outside the liver or both intra- and extrahepatic lesions simultaneously discovered or sequentially detected within a 3-month period. Overall, 6 patients were lost during the follow-up period, and the median follow-up period for these patients was 25.5 months (range, 4.0-240.1 months). The remaining patients were followed up until death or the end of this study.

### Statistical analysis

Statistical analyses were carried out using the statistical software SPSS 13.0 (SPSS, Inc., Chicago, IL, USA) for Windows. The outcome measures included recurrence-free survival (RFS) and overall survival (OS). RFS was calculated from the date of LR to the date of detected recurrence. The status of CRC recurrence was recorded at the date of last follow-up for patients who were lost to follow-up or died of other diseases, or the end of this study. OS was measured from the date of LR to the date of death. Categorical clinicopathologic features were assessed with chi-square or Fisher's exact test. The Cox regression proportional hazards model was used to identify factors influencing RFS, and all significant prognostic factors determined in univariate analysis were then selected for multivariate analysis. The survival curves were constructed using the Kaplan-Meier method, and the curves were compared using the log-rank test. A *p *value of < 0.05 was defined as statistically significant.

## Results

### Demographics and clinicopathologic characteristics

The median follow-up period for the study patients was 27.2 months (range, 0.8-269.2 months). During follow-up, 210 patients (75.3%) developed CRC recurrence, and the median time of recurrence was 9.0 months (range, 1.1-129.8 months). Overall, at the end of this study, 191 patients (68.5%) had died, 26 (9.3%) were alive with CRC, and 62 (22.2%) were alive without evidence of CRC. Table 1 summarizes and compares the clinicopathologic characteristics of the patients in era 1 and era 2. There were significant differences in the age distribution, tumor number, CRC recurrence, and perioperative chemotherapy between the 2 groups. Patients in era 2 were older, had a higher percentage of multiple hepatic metastases (44.4% in era 2 *vs*. 24.2% in era 1; *p *< 0.001), had a lower rate of CRC recurrence (69.5% *vs*. 82.0%; *p *= 0.008), and received perioperative chemotherapeutic regimens different from those in era 1 (*p *< 0.0001). Further detailed analysis regarding chemotherapies showed that a higher percentage of patients in era 2 received fluorouracil and leucovorin combined with irinotecan and oxaliplatin, while patients of era 1 were mainly administered fluorouracil and leucovorin for adjuvant chemotherapy. Notably, 8 (5.3%) patients who were initially considered unsuitable for LR became eligible for surgical resection after chemotherapy in era 2. There was no significant difference in the surgical mortality between the 2 groups.

**Table 1:** Clinicopathological characteristics of patients undergoing LR for hepatic metastasis from CRC in different eras.

Characteristics	Era 1 *n *= 128 (%)	Era 2 *n *= 151 (%)	*p *value
Age in years, median (range)	59 (21-81)	63 (29-88)	0.009
Gender			0.194
Male	76 (59.4)	101 (66.9)	
Female	52 (40.6)	50 (33.1)	
Primary tumor location			0.112
Colon	65 (50.8)	91 (60.3)	
Rectum	63 (49.2)	60 (39.7)	
Liver metastasis			
CEA (mg/dL), median (range)	15.5 (0.5-7025)	12.4 (0.8-4280)	0.743
Metastatic type			0.471
Synchronous	85 (66.4)	94 (62.3)	
Metachronous	43 (33.6)	57 (37.7)	
Tumor number			< 0.0001
Solitary	97 (75.8)	84 (55.6)	
Multiple	31 (24.2)	67 (44.4)	
Maximum tumor size (cm)			0.331
< 5	108 (85.7)	122 (81.3)	
≥5	20 (14.3)	29 (18.7)	
Extent of liver resection			0.209
< 3 segments	88 (68.8)	114 (75.5)	
≥ 3 segments	40 (31.2)	37 (24.5)	
Perioperative chemotherapy			< 0.0001
FU or with LV	69 (54.0)	8 (5.3)	
FU/LV/oxaliplatin	9 (7.0)	36 (23.8)	
FU/LV/irinotecan	9 (7.0)	67 (44.4)	
Other regimens	22 (17.2)	30 (19.9)	
No	19 (14.8)	10 (6.6)	
CRC recurrence			0.008
IHR	57 (44.5)	43 (28.4)	
SR	48 (37.5)	62 (41.1)	
No	23 (18.0)	46 (30.5)	
Resection of CRC recurrence	10 (9.5)*	31 (29.5)*	0.003
Surgical mortality	2 (1.6)	2 (1.3)	1.000

LR, liver resection; CRC, colorectal cancer; CEA, carcinoembryonic antigen; FU, fluorouracil; LV, leucovorin; IHR, intrahepatic recurrence; SR, systemic recurrence; * represents percentage among CRC recurrence.

### Prognostic factors affecting CRC recurrence

Among the patients who developed CRC recurrence, 100 (47.6%) had IHR and 110 (52.4%) had SR according to the classification of recurrence patterns. In era 1, 57 (44.5%) patients had IHR and 48 (37.5%) patients had SR, while in era 2, 43 (28.4%) patients had IHR and 62 (41.1%) patients had SR. Table 2 provides an analysis of the risk factors for CRC recurrence according to the different eras. Univariate analysis showed that the type of hepatic metastases and number of metastatic tumors were significant factors in era 1. Subsequently, multivariate regression analysis of the 2 factors showed that multiple metastatic tumors (*p *= 0.035; hazard ratio [HR] = 1.6) was the sole independent risk factor affecting CRC recurrence in era 1. In era 2, 4 factors, including the patient's gender, type of hepatic metastases, number of metastatic tumors, and width of negative resection margin, were identified by univariate analysis. Multivariate regression analysis of these factors indicated that multiple metastatic tumors (*p *= 0.017; HR = 1.6) and resection margin < 0.5 cm (*p *= 0.011; HR = 1.7) significantly influenced the recurrence of CRC in era 2. Further analysis of the clinical prognostic factors for all patients showed that the presence of multiple metastatic tumors and synchronous metastases were independent risk factors affecting CRC recurrence (Table 3).

**Table 2:** Univariate and multivariate analyses of clinicopathological factors affecting CRC recurrence of patients after liver resection for hepatic metastases in different eras.

Factors	Era 1				Era 2			
	
	Univariate			Multivariate	Univariate			Multivariate
	
	*n*	Medium RFS months (95%CI)	*p *value	*p *value	*n*	Medium RFS months (95%CI)	*p *value	*p *value
Age (years)								
< 65	89	8.0 (6.9-9.0)	0.296	-	84	12.0 (8.3-15.7)	0.300	-
≥65	39	12.0 (9.7-14.3)			67	21.0 (16.4-25.7)		
Gender								
Female	52	10.0 (8.1-11.9)	0.274	-	50	24.4 (11.7-37.1)	0.014	0.218
Male	76	9.0 (7.5-10.4)			101	14.5 (10.1-18.9)		
Primary tumor								
Colon	65	11.0 (8.9-13.0)	0.116	-	91	17.5 (10.4-24.6)	0.961	-
Rectum	63	8.0 (6.1-9.9)			60	16 (6.6-25.3)		
Serum CEA (mg/dL)								
≤200	83	11.0 (8.8-13.1)	0.550	-	115	17.5 (11.0-24.0)	0.362	-
> 200	38	9.0 (7.8-10.1)			94	15.3 (6.5-24.0)		
Metastatic type								
Synchronous	85	8.0 (6.8-9.2)	0.052	0.075	94	12.9 (8.5-17.3)	0.009	0.076
Metachronous	43	11.0 (8.8-13.1)			57	21.0 (7.4-34.7)		
Tumor number								
Solitary	97	10.0 (8.8-11.1)	0.022	0.035	84	22.1 (16.5-27.8)	0.001	0.028
Multiple	31	7.0 (5.3-8.7)		1.6 (1.0-2.5)	67	12.0 (10.6-13.5)		1.6 (1.1-2.4)
Maximum tumor size								
< 5 cm	108	10.0 (8.8-11.2)	0.939	-	122	18.0 (12.6-23.5)	0.162	-
≥5 cm	20	8.0 (3.1-2.9)			29	11.9 (6.9-16.8)		
Liver resection extent								
< 3 segments	88	10.0 (8.5-11.4)	0.743	-	114	17.5 (11.1-23.9)	0.257	-
≥3 segments	40	9.0 (7.6-10.4)			37	13.0 (6.0-19.9)		
Resection margin (cm)								
< 0.5	60	9.0 (7.5-10.4)	0.773	-	87	12.0 (10.3-13.7)	0.001	0.011
≥0.5	68	10.0 (8.0-11.9)			64	23.3 (16.9-29.6)		1.7 (1.1-2.6)
Histology grade								
Low grade	116	10.0 (8.8-11.2)	0.101	-	145	16.5 (11.0-22.0)	0.921	-
High grade	12	5.0 (2.1-7.9)			6	5.3 (0.0-30.4)		

RFS, recurrence-free survival; CRC, colorectal cancer; CEA, carcinoembryonic antigen; CI, confidence interval

**Table 3:** Univariate and multivariate analyses of clinicopathological factors affecting CRC recurrence in all patients after liver resection for hepatic metastases.

Factors	Univariate analysis			Multivariate analysis
	*n*	Medium RFS months (95% CI)	*p *value	*p *value (HR, 95% CI)
Age (years)				
< 65	173	10.0 (8.4-11.6)	0.103	-
≥65	106	16.9 (9.9-24.0)		
Gender				
Male	177	12.0 (7.7-16.3)	0.021	0.134
Female	102	11.0 (9.0-13.0)		
Primary tumor				
Colon	156	12.9 (8.5-17.2)	0.234	-
Rectum	123	10.0 (8.5-11.5)		
Serum CEA (mg/dL)				
< 200	198	12.0 (10.6-13.4)	0.567	-
≥200	72	10.0 (7.3-12.7)		
Metastatic type				
Synchronous	179	10.8 (9.3-12.3)	0.002	0.014
Metachronous	100	15.0 (9.1-20.9)		1.5 (1.1-2.0)
Tumor number				
Solitary	181	14.0 (8.9-19.1)	0.001	0.028
Multiple	98	10.0 (7.8-8.7)		1.4 (1.0-1.9)
Maximum tumor size				
< 5 cm	230	12.0 (10.0-14.0)	0.420	-
≥5 cm	49	10.0 (6.9-13.1)		
Extent of liver resection				
< 3 segments	202	12.0 (9.3-14.7)	0.235	-
≥3 segments	77	10.0 (7.9-12.1)		
Resection margin (cm)				
< 0.5	147	10.0 (8.5-11.5)	0.019	0.073
≥0.5	132	14.5 (9.1-19.9)		
Histologic grade				
Low grade	261	12.0 (9.3-14.7)	0.184	-
high grade	18	5.3 (1.8-8.8)		

RFS, recurrence free survival; CRC, colorectal cancer; CEA, carcinoembryonic antigen; CI, confidence interval; HR, hazard ratio

### Outcomes after LR of hepatic metastasis

**Figure 1:**
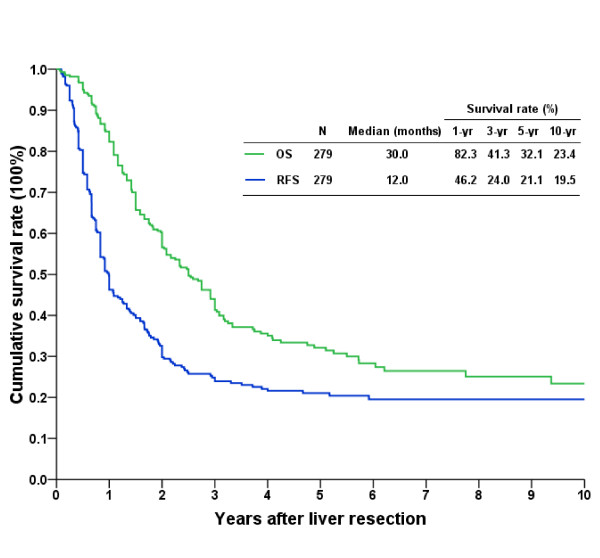
**Long-term cumulative recurrence-free survival (RFS) and overall survival (OS) curves of patients undergoing liver resection for hepatic metastasis from colorectal cancer**.

**Figure 2:**
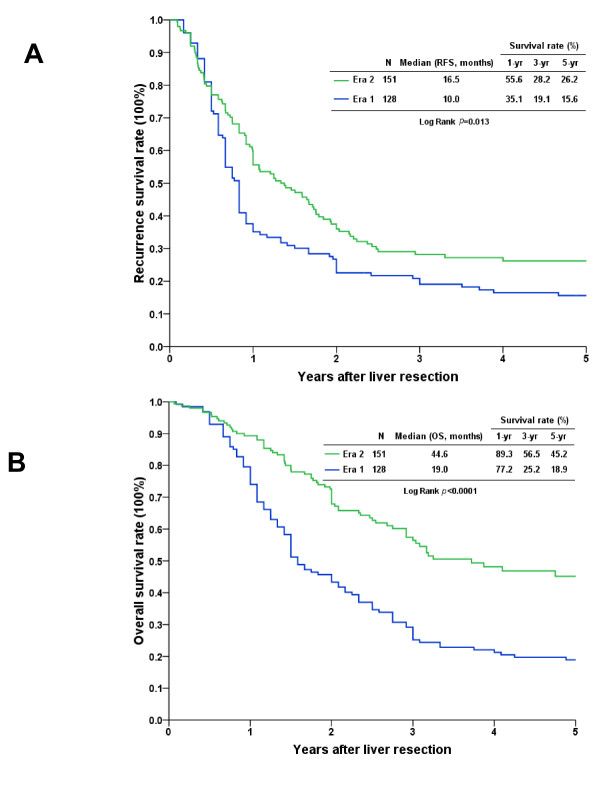
**Cumulative survival curves of patients who underwent liver resection for hepatic metastasis according to the era of their treatment**. **A**. The recurrence-free survival (RFS) rates in era 2 were significantly better than the RFS rates in era 1 (*p *= 0.013). **B**. The overall survival (OS) rate in era 2 was better than the OS rate in era 1 (*p *< 0.0001).

Overall, the 3-, 5-, and 10-year RFS rates were 24.0%, 21.1%, and 19.5%, respectively, and the 3-, 5-, and 10-year OS rates were 41.3%, 32.1%, and 23.4%, respectively (Figure 1). Upon further analyses of the survival rates of patients who received LR for hepatic metastasis according to the era in which they occurred, we found that the outcome of patients in era 2 was significantly better than that of patients in era 1 (Figure 2). Era 2 patients had better RFS curves, and the 1-, 3-, and 5-year RFS rates were 55.6%, 28.2%, and 26.2%, respectively, with a median time of CRC recurrence of 16.5 months. The 1-, 3-, and 5-year RFS rates in era 1 patients were 35.1%, 19.1%, and 15.6%, respectively, with a median time of CRC recurrence of 10.0 months (Figure 2A; *p *= 0.013). The 1-, 3-, and 5-year OS rates of the patients in era 2 (89.3%, 56.5%, and 45.2%, respectively, with a median survival of 44.6 months) were better than those of patients in era 1 (77.2%, 25.2%, and 18.9%, respectively, with a median survival of 19 months) (Figure 2B; *p *< 0.0001).

### CRC recurrence after LR

**Figure 3:**
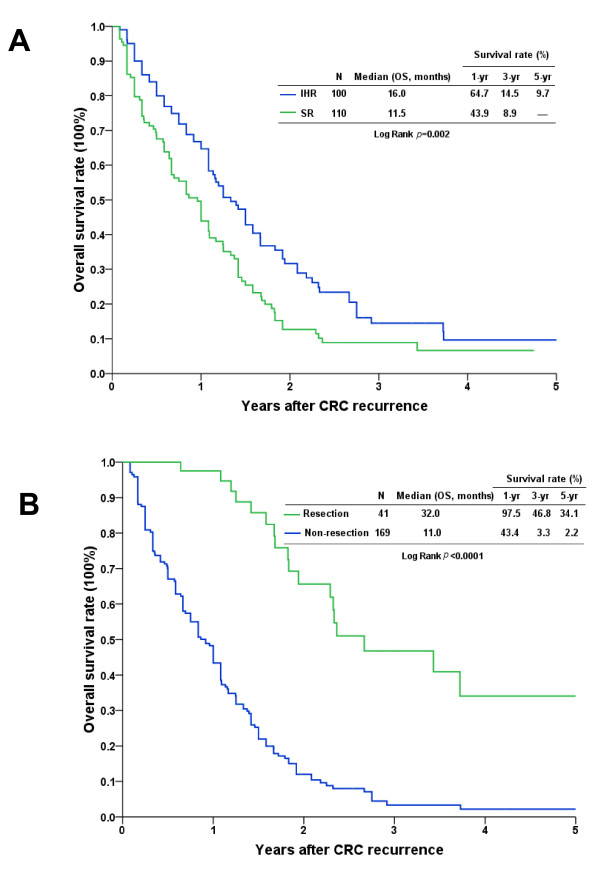
**Kaplan-Meier cumulative survival curves of patients who underwent liver resection for hepatic metastasis after colorectal cancer recurrence**. **A**. The survival curve of patients with intrahepatic recurrence (IHR) was relatively better than that of patients with systemic recurrence (SR) (*p *= 0.002). **B**. Patients who had undergone surgical resection of recurrent lesions had a better survival curve than did patients without resection (*p *< 0.0001).

The cumulative survival rates from the time of identification of CRC recurrence after LR were further analyzed, and patients with IHR and SR had poor survival curves. Nonetheless, patients with IHR had a relatively better survival curve than patients with SR. The 3-year survival rates were 14.5% in patients with IHR and 8.9% in patients with SR (Figure 3A; *p *= 0.002). Of the 210 patients who developed CRC recurrence after LR, 41 (19.5%), including 24 with IHR and 17 with SR, underwent surgical resection for the recurrent tumor. Of these patients, 31 (29.5%) had CRC recurrence in era 2 and 10 (9.5%) had CRC recurrence in era 1. The comparison showed that the ratio of patients who underwent surgical resection for CRC recurrence was significantly higher in era 2 than in era 1 (Table 1; *p *= 0.003).

The survival rates according to whether or not patients received surgical resection for recurrent CRC were further analyzed. The results demonstrated that the outcome of patients who underwent surgical resection (3- and 5-year survivals of 46.8% and 34.1%, respectively) was significantly better than that of patients who had no resection (3- and 5-year survivals of 3.3% and 2.2%, respectively; Figure 3B; *p *< 0.0001). The median follow-up period after surgical resection for recurrent CRC for these patients was 22.7 months (range, 4.2-107.5 months). During the follow-up period, 18 patients died of CRC relapse, 11 were still alive with cancer, and the remaining 12 were alive and cancer-free at the end of this study.

## Discussion

Although CRC is a common malignancy worldwide, it has only recently been ranked as a leading cancerous disease in East Asian countries. Therefore, the concept of LR for CRC hepatic metastasis has started to receive considerable attention in East Asian countries. In this study, we have shown that the outcome of patients with CRC hepatic metastasis who have undergone LR has notably improved since 2003. The number of patients who have undergone LR for CRC hepatic metastasis has also shown a remarkable increase.

The overall recurrence incidence of 75.3% after LR for CRC hepatic metastasis found in our study is slightly higher than the recurrence rates reported by others (range, 40-74%) [12-14]. However, our study evaluated patients treated over a 20-year period, and treatment strategies for CRC hepatic metastasis have changed along with advances in systemic therapy. As we had selected an arbitrary break point around the year 2003 on the basis of access to modern chemotherapeutic regimens including oxaliplatin and irinotecan at our institute, both the 5-year RFS and the incidence of recurrence in era 2 were significantly better than that in era 1. The percentage of patients who received modern chemotherapeutic regimens was significantly higher in era 2, suggesting that the use of current chemotherapy is crucial for the improvement in patient outcomes over the years. However, the regimens of adjuvant chemotherapy in our patients were not identical, and comparison of patients grouped by different chemotherapeutic regimens was not practical because of the limited number of patients in each group. Therefore, further information to clarify the effect of a specific protocol in terms of postoperative adjuvant chemotherapy regimens on patient outcomes is required.

A number of previous reports have shown several prognostic factors that predict the outcome of patients undergoing LR for CRC hepatic metastasis, and similar factors were noted in our study [4,9,10]. The presence of multiple hepatic metastases is the sole prognostic factor influencing the risk of CRC recurrence in both eras, but the width of the negative resection margin appeared to be an additional risk factor during the recent era. The data generated from this analysis cannot completely explain the statistical differences. However, patients in era 2 have higher ratios of multiple hepatic metastases than those in era 1, and it is somewhat difficult to achieve an adequate distance of negative resection margin during LR for such patients. In particular, to maintain an adequate remnant liver volume, limited LR or enucleation of multiple tumors might be performed, and this might lead to inadequate safe resection margins. Therefore, the width of the negative resection margin as a prognostic factor might be explained, at least in part, by aggressive LR for patients with multiple metastases in era 2. Nonetheless, the advantages and disadvantages of adequate safe resection margins remain controversial [15-18]. Despite the debate concerning the width of a negative resection margin, there is consensus that difficulty in obtaining an adequate resection margin should not be used as an exclusion criterion for LR.

The concept of managing CRC hepatic metastasis by surgical resection has greatly evolved in the last decade. For example, the traditional indications of LR for CRC hepatic metastases before the year 2000 were mainly limited to metastases confined to 1 lobe and/or less than 4 metastases,[19] which were not considered exclusion criteria for LR afterward. The advancement in surgical techniques and instruments used for LR has also decreased the perioperative morbidity and mortality, leading to a more aggressive approach of using LR for multiple hepatic metastases from CRC over time. Confidence in the utility of LR is also reflected in the increased age of patients in era 2 than in era 1. Moreover, increasing evidence shows that surgical resection of metastatic lesions with curative intent has now become a standard practice for dealing with several malignancies,[20-22] and the policy has been expanded to include patients with recurrence after LR for CRC metastasis. Although it remains arguable that the prognosis of patients who are suitable to undergo surgical resection is naturally better than that of patients who are unable to undergo surgical resection, an aggressive attitude with regard to surgical resection is indeed a benefit to selected patients who have CRC hepatic metastasis or recurrent CRC from LR for hepatic metastasis. Similar to other recent studies,[23,24] we also found surgical resection of isolated recurrent lesions in selected patients to be of advantage to those who have undergone LR for CRC hepatic metastasis.

## Conclusions

In summary, despite the study limitation imposed by the relatively small number of patients and the retrospective nature, we showed that aggressive LR for CRC hepatic metastasis and a combination of current chemotherapeutic regimens led to improvements in the long-term outcome of such patients. However, the ultimate aim is to establish and standardize a promising treatment protocol that involves the development of novel systemic chemotherapy regimens for neoadjuvant and postoperative adjuvant treatments, combined with aggressive surgical resection to effectively prolong survival or even cure the patient of CRC hepatic metastasis.

## Competing interests

The authors declare that they have no competing interests.

## Authors' contributions

Chan KM participated in the design of the study and drafted the manuscript. Chiang JM, Lee CF, and Yu MC participated in the acquisition of data. Lee WC, Chen JS, and Wang JY participated in the design and coordination of the study. All authors read and approved the final manuscript.

## References

[B1] Manfredi S Lepage C Hatem C Coatmeur O Faivre J Bouvier AM Epidemiology and management of liver metastases from colorectal cancer Ann Surg 2006 244 254 259 10.1097/01.sla.0000217629.94941.cf 16858188 PMC1602156

[B2] Simmonds PC Primrose JN Colquitt JL Garden OJ Poston GJ Rees M Surgical resection of hepatic metastases from colorectal cancer: a systematic review of published studies Br J Cancer 2006 94 982 999 10.1038/sj.bjc.6603033 16538219 PMC2361241

[B3] Garden OJ Rees M Poston GJ Mirza D Saunders M Ledermann J Primrose JN Parks RW Guidelines for resection of colorectal cancer liver metastases Gut 2006 55 Suppl 3 1 8 16835351 10.1136/gut.2006.098053 PMC1860000

[B4] Rees M Tekkis PP Welsh FK O'Rourke T John TG Evaluation of long-term survival after hepatic resection for metastatic colorectal cancer: a multifactorial model of 929 patients Ann Surg 2008 247 125 135 10.1097/SLA.0b013e31815aa2c2 18156932

[B5] Kato T Yasui K Hirai T Kanemitsu Y Mori T Sugihara K Mochizuki H Yamamoto J Therapeutic results for hepatic metastasis of colorectal cancer with special reference to effectiveness of hepatectomy: analysis of prognostic factors for 763 cases recorded at 18 institutions Dis Colon Rectum 2003 46 S22 S31 14530655 10.1097/01.DCR.0000089106.71914.00

[B6] Wei AC Greig PD Grant D Taylor B Langer B Gallinger S Survival after hepatic resection for colorectal metastases: a 10-year experience Ann Surg Oncol 2006 13 668 676 10.1245/ASO.2006.05.039 16523369

[B7] Fernandez FG Drebin JA Linehan DC Dehdashti F Siegel BA Strasberg SM Five-year survival after resection of hepatic metastases from colorectal cancer in patients screened by positron emission tomography with F-18 fluorodeoxyglucose (FDG-PET) Ann Surg 2004 240 438 447 10.1097/01.sla.0000138076.72547.b1 15319715 PMC1356434

[B8] Choti MA Sitzmann JV Tiburi MF Sumetchotimetha W Rangsin R Schulick RD Lillemoe KD Yeo CJ Cameron JL Trends in long-term survival following liver resection for hepatic colorectal metastases Ann Surg 2002 235 759 766 10.1097/00000658-200206000-00002 12035031 PMC1422504

[B9] Fong Y Fortner J Sun RL Brennan MF Blumgart LH Clinical score for predicting recurrence after hepatic resection for metastatic colorectal cancer: analysis of 1001 consecutive cases Ann Surg 1999 230 309 318 10.1097/00000658-199909000-00004 10493478 PMC1420876

[B10] Nordlinger B Guiguet M Vaillant JC Balladur P Boudjema K Bachellier P Jaeck D Surgical resection of colorectal carcinoma metastases to the liver. A prognostic scoring system to improve case selection, based on 1568 patients. Association Francaise de Chirurgie Cancer 1996 77 1254 1262 10.1002/(SICI)1097-0142(19960401)77:7<1254::AID-CNCR5>3.0.CO;2-I 8608500

[B11] Ueno H Mochizuki H Hashiguchi Y Hatsuse K Fujimoto H Hase K Predictors of extrahepatic recurrence after resection of colorectal liver metastases Br J Surg 2004 91 327 333 10.1002/bjs.4429 14991634

[B12] Bozzetti F Doci R Bignami P Morabito A Gennari L Patterns of failure following surgical resection of colorectal cancer liver metastases. Rationale for a multimodal approach Ann Surg 1987 205 264 270 10.1097/00000658-198703000-00008 3827362 PMC1492706

[B13] Holm A Bradley E Aldrete JS Hepatic resection of metastasis from colorectal carcinoma. Morbidity, mortality, and pattern of recurrence Ann Surg 1989 209 428 434 10.1097/00000658-198904000-00007 2930288 PMC1493991

[B14] Sugihara K Hojo K Moriya Y Yamasaki S Kosuge T Takayama T Pattern of recurrence after hepatic resection for colorectal metastases Br J Surg 1993 80 1032 1035 10.1002/bjs.1800800837 8402060

[B15] Bodingbauer M Tamandl D Schmid K Plank C Schima W Gruenberger T Size of surgical margin does not influence recurrence rates after curative liver resection for colorectal cancer liver metastases Br J Surg 2007 94 1133 1138 10.1002/bjs.5762 17514637

[B16] Nuzzo G Giuliante F Ardito F Vellone M Giovannini I Federico B Vecchio FM Influence of surgical margin on type of recurrence after liver resection for colorectal metastases: a single-center experience Surgery 2008 143 384 393 10.1016/j.surg.2007.09.038 18291260

[B17] Pawlik TM Scoggins CR Zorzi D Abdalla EK Andres A Eng C Curley SA Loyer EM Muratore A Mentha G Effect of surgical margin status on survival and site of recurrence after hepatic resection for colorectal metastases Ann Surg 2005 241 715 22 discussion 10.1097/01.sla.0000160703.75808.7d 15849507 PMC1357126

[B18] Muratore A Ribero D Zimmitti G Mellano A Langella S Capussotti L Resection margin and recurrence-free survival after liver resection of colorectal metastases Ann Surg Oncol 2010 17 1324 1329 10.1245/s10434-009-0770-4 19847565

[B19] Rees M Plant G Bygrave S Late results justify resection for multiple hepatic metastases from colorectal cancer Br J Surg 1997 84 1136 1140 10.1002/bjs.1800840828 9278662

[B20] Fourquier P Regnard JF Rea S Levi JF Levasseur P Lung metastases of renal cell carcinoma: results of surgical resection Eur J Cardiothorac Surg 1997 11 17 21 10.1016/S1010-7940(96)01013-5 9030784

[B21] van Geel AN Pastorino U Jauch KW Judson IR van CF Buesa JM Nielsen OS Boudinet A Tursz T Schmitz PI Surgical treatment of lung metastases: The European Organization for Research and Treatment of Cancer-Soft Tissue and Bone Sarcoma Group study of 255 patients Cancer 1996 77 675 682 10.1002/(SICI)1097-0142(19960215)77:4<675::AID-CNCR13>3.0.CO;2-Y 8616759

[B22] Ollila DW Complete metastasectomy in patients with stage IV metastatic melanoma Lancet Oncol 2006 7 919 924 10.1016/S1470-2045(06)70938-X 17081917

[B23] Petrowsky H Gonen M Jarnagin W Lorenz M DeMatteo R Heinrich S Encke A Blumgart L Fong Y Second liver resections are safe and effective treatment for recurrent hepatic metastases from colorectal cancer: a bi-institutional analysis Ann Surg 2002 235 863 871 10.1097/00000658-200206000-00015 12035044 PMC1422517

[B24] Adam R Bismuth H Castaing D Waechter F Navarro F Abascal A Majno P Engerran L Repeat hepatectomy for colorectal liver metastases Ann Surg 1997 225 51 60 10.1097/00000658-199701000-00006 8998120 PMC1190605

